# Enhanced Bioactive Compounds and Antioxidant Activity in Germinated Seeds of the New Peanut Variety

**DOI:** 10.3390/biotech14010012

**Published:** 2025-02-25

**Authors:** Hwan-Hee Yu, Jong-Suk Park, Sanghyun Lee

**Affiliations:** 1 Department of Plant Science and Technology, Chung-Ang University, Anseong 17546, Republic of Korea; hhy120659@cau.ac.kr; 2 Jeonbuk State Agricultural Research & Extension Services, Iksan 54591, Republic of Korea; jspark88@korea.kr; 3 Natural Product Institute of Science and Technology, Anseong 17546, Republic of Korea

**Keywords:** Sinpalkwang, *trans*-resveratrol, soyasaponin Bb, HPLC method, peanut

## Abstract

The sprout market in Korea is expanding as consumers seek healthier food options and farmers strive to increase added value and competitiveness. This study examined the changes in the phytochemical composition of Sinpalkwang (SPK), a peanut variety developed in Korea, during germination. Four samples (SPK1, SPK2, SPK3, and SPK4) were collected at different growth stages and analyzed for total polyphenol content (TPC), total flavonoid content (TFC), and antioxidant activities using ABTS^+^ and DPPH assays. The levels of *trans*-resveratrol and soyasaponin Bb were quantified using high-performance liquid chromatography (HPLC) with a photo-diode array (PDA). Among the samples, SPK2 exhibited the highest TFC (1.61 mg QE/g ext.) and *trans*-resveratrol content (0.054 mg/g ext.), while SPK4 showed the highest TPC (29.38 mg TAE/g ext.) and soyasaponin Bb content (6.543 mg/g ext.). In terms of radical scavenging activities, SPK2 and SPK3 performed best in the ABTS^+^ and DPPH assays, respectively. Germinated samples demonstrated strong results across all analyses, highlighting the benefits of germination in enhancing phytochemical properties. This study provides foundational information on the phytochemical composition of SPK and the effects of germination. Future research will focus on optimizing germination conditions to further enhance the functionality and value of this Korean-bred variety as a source of high-value bioactive ingredients.

## 1. Introduction

Peanuts (*Arachis hypogaea* L.) are a member of the Leguminosae family and are consumed worldwide due to their rich nutrients, including protein, fatty acid, minerals, vitamins, and phytochemicals. Peanuts are cultivated in Asia, Africa, and the Americas, serving as versatile ingredients in cooking, snacks, spreads, butter, and oils [1,2]. Phytochemicals in peanuts, such as carotenoids, stilbene resveratrol, proanthocyanidins, and catechins, have been shown to offer significant health benefits even in small amounts [2]. Numerous studies have demonstrated their efficacy, and some are now commercially produced through artificial synthesis. However, consuming naturally occurring phytochemicals is considered safer due to the reduced risk of side effects or toxicity [3].

Germination is a straightforward and effective method to enhance phytochemical content. During germination, phytochemical accumulation increases, leading to improved physiological activity [4,5]. Studies on legume germination also indicate that it reduces antinutrient compounds, which inhibit nutrient absorption [6]. Germination is eco-friendly, requiring only clean water, no pesticides, and a short growth period [7].

In Korea, the growing interest in health has popularized the consumption of various sprout seeds, including kohlrabi, red radish, broccoli, cabbage, radish sprouts, peas, lentils, and mung beans, as food ingredients. These sprouts also hold high commercial value as natural functional ingredients and highly bioactive food materials. Peanut sprouts, primarily processed into powder form, are used in products like rice cakes and baked goods. A study on madeleines containing peanut sprout powder revealed that increasing the amount of peanut sprout powder resulted in higher total polyphenol content, total flavonoid content, and antioxidant activity [8].

Sinpalkwang (SPK) is a high-yield peanut variety developed through breeding in Korea. It was developed via the cross-fertilization of the large-grain Palkwang and the short-stemmed PI156649 varieties by the National Institute of Crop Science, Jeonbuk, Republic of Korea [9]. SPK is characterized by large grains and short stems [9]. SPK has lower crude fat and higher linoleic acid compared to Daekwang, which was previously cultivated in Korea. Selected as a new variety in 2012, SPK continues to be widely cultivated in Korea [9]. Despite ongoing efforts to develop new varieties through breeding, research on peanut varieties grown in Korea remains limited. In the case of SPK, studies on its phytochemical composition are scarce, primarily focusing on *trans*-resveratrol and soyasaponin Bb.

*trans*-Resveratrol, a polyphenol found in grape skins, red or purple fruits, peanuts, and wine, has gained significant attention as a potential factor in the “French paradox”, where the French exhibit a low incidence of cardiovascular disease despite a diet high in saturated fats [10,11]. This compound not only possesses strong antioxidant properties but also promotes cardiovascular health [10]. However, the oral bioavailability of *trans*-resveratrol is limited; thus, recent studies have explored the use of nanoparticles, such as solid lipid nanoparticles, to enhance its delivery to target organs [12,13]. In addition, *trans*-resveratrol has been investigated as an activator of Sirtuin 1 (SIRT1), which regulates the post-translational modifications of proteins, contributing to the longevity of cells [14,15]. At NAD^+^ concentrations above 100 µM, *trans*-resveratrol can increase SIRT1 activity eightfold by reducing the K_M_ of the deacetylation reaction [16,17]. SIRT1 activation through either resveratrol or calorie restriction consequently improves hepatic insulin sensitivity and regulates cholesterol and fatty acid metabolism in the liver [18].

Soyasaponin is a component that exists in many legume crops regardless of species [19]. Soyasaponins, which consist of aglycone and oligosaccharide groups, can be categorized into A, B, and E types based on their aglycone structure [20]. Soyasaponin A contains soyasapogenol A as its aglycone. Similarly, soyasaponin B and E have soyasapogenol B and E as their respective aglycones [21]. Soyasaponin Bb is the main monomeric compound of soyasaponins [22]. Their type and content vary between seed species, growth conditions, and growth stage [23]. Processing methods also impact soyasaponin levels, which are lower during boiling and higher when soaking or sprouting [23]. In particular, soybean sprouts have been shown to raise soyasaponin levels 3.2-fold compared to mature soybeans because the plant synthesizes soyasaponins as a defense mechanism during germination [24]. Higher soyasaponin levels are associated with stronger antioxidant efficacy, and at higher concentrations, they exert an inhibitory effect on the growth and survival of colon cancer cells [25]. Additionally, soyasaponins help prevent fat accumulation in the liver and reduce adipose tissue by promoting fecal excretion [26].

The present study aimed to evaluate the content of bioactive compounds in SPK using HPLC-PDA and their relationship with antioxidant efficacy, with a focus on *trans*-resveratrol and soyasaponins. Since SPK is a high-yielding variety, it will have high nutritional value if the time when bioactive compounds are most active is identified. This also yield beneficial potential for humans, as it has a higher antioxidant effect induced by enhanced bioactive compounds.

## 2. Materials and Methods

### 2.1. Plant Materials

**Figure 1:**
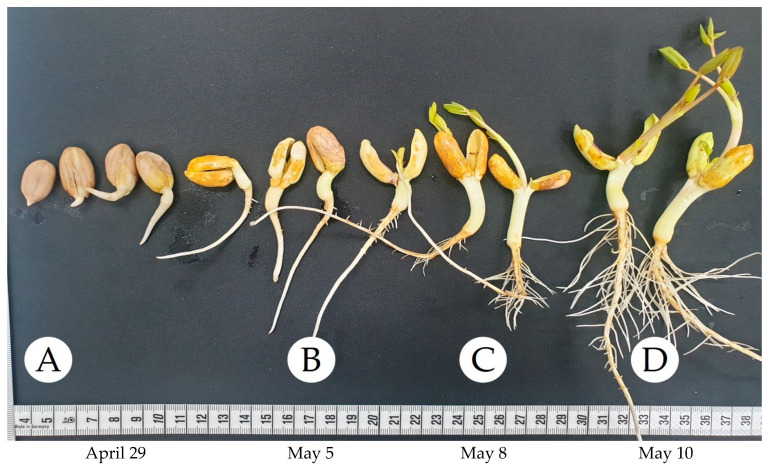
SPK samples by sampling time: (**A**) SPK 1, (**B**) SPK 2, (**C**) SPK 3, and (**D**) SPK 4.

As shown in Figure 1, SPK was classified into four serial growth stages. SPK 1 is the dried SPK peanut. SPK 2 is the stage where the sprouts are not visible outside the cotyledons, while SPK 3 has sprouts that are visible about 1 cm outside the cotyledons. In SPK 4, the sprouts are visible about 3 cm outside the cotyledons. All samples were provided by Jeonbuk State Agricultural Research, Iksan, Republic of Korea. Voucher specimens were stored in Jeonbuk State Agricultural Research, Iksan, Republic of Korea. After collecting, the samples (voucher No. LEE2024-0428) were analyzed in powdered form in July 2024.

### 2.2. Growth Conditions

Peanut seeds of the SPK variety were soaked in water for 24 h on 28 April 2024. Subsequently, the seeds were transferred to a cultivation tray and subjected to aeroponic cultivation. The spray system was programmed to spray water for 30 s at 10 min intervals. The aeroponics procedure was conducted entirely in darkness and continued until 10 May 2024.

### 2.3. Instruments and Reagents

**Figure 2:**
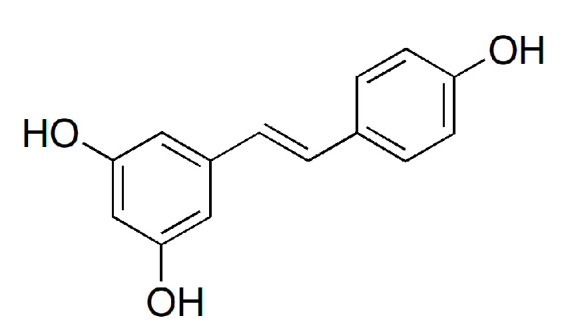
Chemical structure of *trans*-resveratrol (**1**).

**Figure 3:**
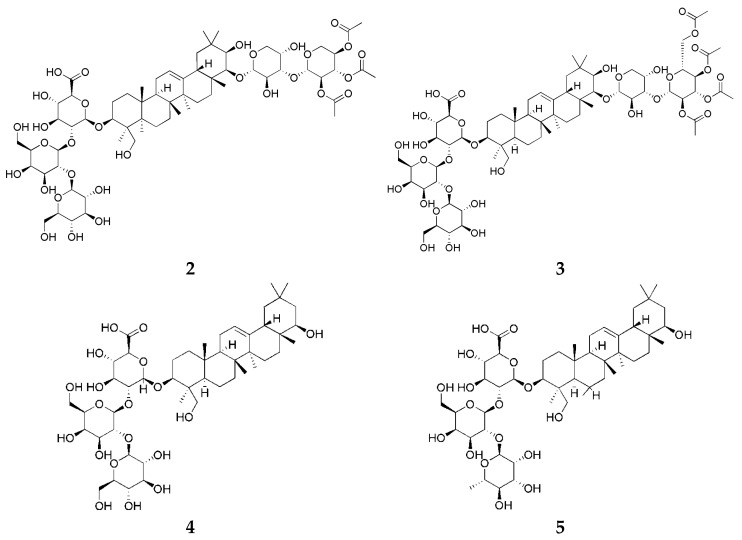
Chemical structures of soyasaponins Aa (**2**), Ab (**3**), Ba (**4**), and Bb (**5**).

*trans*-Resveratrol and soyasaponins were analyzed using HPLC (Waters Alliance system e2695 Separations Module, Milford, MA, USA) with a 2998 PDA and an INNO C18 column (4.6 × 250 mm, 5 µm). HPLC-grade water (H_2_O) and methanol (MeOH) were purchased from Honeywell (Burdick and Jackson, Muskegon, MI, USA). Ethanol (EtOH) was purchased from Samchun Chemicals (Pyeongtaek, Republic of Korea). Acetonitrile (ACN; HPLC grade) was obtained from J. T. Baker (Phillipsburg, PA, USA). Acetic acid and phosphoric acid (HPLC grade) were purchased from Fisher Scientific (Loughborough, Leicestershire, UK). Polyvinylidene fluoride (PVDF) membrane filter was obtained from Hyundai Micro (Seoul, Republic of Korea). In addition, *trans*-resveratrol (**1**), soyasaponin Aa (**2**), soyasaponin Ab (**3**), soyasaponin Ba (**4**), and soyasaponin Bb (**5**) were supplied by Natural Product Institute of Science and Technology (www.nist.re.kr; accessed on 5 July 2024), Anseong, Republic of Korea (Figure 2 and Figure 3).

### 2.4. Preparation of SPK Extracts

Dried SPK samples were ground into a powder, mixed with 240 mL of 95.0% EtOH, and heated in a reflux condenser for 3 h. The ratio of dry sample to solvent was set at 1:40. The solution extracted from the sample was poured onto filter paper to remove the solid matter. The filtered solution was then concentrated using a rotary evaporator. The dried solid-state extracts were used to prepare samples for subsequent experiments.

### 2.5. Sample Preparation for Antioxidant Activities

The dried extracts of SPK 1, SPK 2, SPK 3, and SPK 4 (50 mg each) were individually sonicated together with 50% MeOH for 40 min until they were completely dissolved. The solutions were centrifuged for 10 min, and the supernatant was collected and filtered through a PVDF membrane filter with a pore size of 0.45 µm. Antioxidant efficacy was assessed based on IC_50_, which is the concentration required to reduce 2,2′-azino-bis-(3-ethylbenzothiazoline-6-sulfonic) acid (ABTS^+^) and 2,2-diphenyl-1-picrylhydrazyl (DPPH) radicals by 50% [27].

### 2.6. ABTS^+^ Radical Scavenging Activity of SPK

ABTS^+^ radical scavenging activity was evaluated using a 96-well plate (SPL Life Sciences, Pocheon, Republic of Korea). ABTS^+^ was acquired from Thermo Fisher Scientific Inc. (Waltham, MA, USA). The dried SPK extracts were mixed with 50% MeOH. The ABTS^+^ and ascorbic acid stock solutions (A0278-100G, Sigma-Aldrich, St. Louis, MO, USA) were combined with water and used as the working solutions. The ascorbic acid working solution (10 µL) and the SPK samples (10 µL) were combined in each well, and 200 µL of the ABTS^+^ working solution was added. The reaction was left to occur for 30 min in the dark. The absorbance of the ABTS^+^ solution was measured at 734 nm using a microplate reader (BioTek Epoch, Santa Clara, CA, USA).

### 2.7. DPPH Radical Scavenging Activity of SPK

DPPH radical scavenging activity was performed using a 96-well microplate. DPPH was acquired from Thermo Fisher Scientific Inc. The dried SPK extracts were mixed with 50% MeOH. A stock solution of ascorbic acid was mixed with water and used as a working solution. A DPPH working solution was produced by mixing the DPPH stock solution with 95% MeOH. The ascorbic acid working solution (10 µL) and the individual samples (10 µL) were combined in the wells of the microplate, to which 200 µL of DPPH working solution was subsequently added. The reaction was left to proceed in the dark for 30 min. The absorbance of the DPPH reaction at 514 nm was then measured using a microplate reader.

### 2.8. Total Polyphenol Content (TPC) of SPK

The TPC of the SPK samples was measured using a 96-well plate. Folin and Ciocalteu’s phenol reagent (2N) and tannic acid (ACS reagent) were purchased from Sigma-Aldrich (Saint Louis, MA, USA). Sodium carbonate anhydrous was acquired from Daejung Chemicals and Metals (Siheung, Republic of Korea). The tannic acid was utilized as a standard solution in TPC analysis. Therefore, tannic acid of 5000 ppm was added to water in six concentrations to draw a calibration curve. All of the samples were completely liquefied by sonication and then purified using a 0.45 µm membrane filter. The 7.5% sodium carbonate solution was employed as the interaction solution. After taking 60 µL of each SPK sample, 40 µL of 2N Folin and Ciocalteu’s phenol reagent, and 100 µL of the 7.5% sodium carbonate solution together, the optical density was measured at 760 nm using a microplate reader.

### 2.9. Total Flavonoid Content (TFC) of SPK

The TFC of the SPK samples was measured using a 96-well plate. EtOH, water, 5000 ppm quercetin (Sigma-Aldrich, MA, USA), and 2% aluminum (III) chloride hexahydrate were used for TFC evaluation. The 2% aluminum (III) carbohydrate solution was produced by mixing aluminum chloride hexahydrate (Sigma-Aldrich, MA, USA) and the water. A calibration curve was produced in this way for the TPC using a quercetin solution instead of tannic acid. After adding 100 µL of the 2% aluminum (III) carbohydrate solution to 100 µL of each sample, the mixture was left in the dark for 10 min. The optical density was then measured at 430 nm using a microplate reader.

### 2.10. HPLC Analysis of Trans-Resveratrol

HPLC analysis of *trans*-resveratrol was conducted using a reverse phase column (INNO C_18_ column, 4.6 × 250 nm, Youngjin Biochrom, Seongnam, Republic of Korea) at 35 °C. The standard solution was prepared by mixing the *trans*-resveratrol (**1**) with 80% MeOH to produce a concentration of 1000 ppm (1 mg/mL) and sonicating it for 20 min. The dried SPK extracts were dissolved in 80% MeOH under sonication for 20 min to produce a 50,000 ppm (50 mg/mL) solution. The resulting solution was filtered and diluted based on the HPLC results recorded at 310 nm. All of the solutions were injected at 5 µL each maintained at 35 °C. Mobile phases A and B were water containing 0.5% acetic acid and ACN, respectively, with a flow rate of 1.0 mL/min. The A/B ratio was 85:15 for 5 min, followed by 77:23 for 10 min, before following a gentle gradient to finish at 70:30. After this process, the column was washed with 100% ACN for 15 min. After washing, the A/B ratio was set at 85:15 for 10 min for stabilization.

### 2.11. HPLC Analysis of Soyasaponins Aa, Ab, Ba, and Bb

Soyasaponins Aa, Ab, Ba, and Bb were analyzed using HPLC-PDA with a reverse-phase column (YMC-Pack Pro C18 column, 4.6 × 250 nm, 5 µm, YMC Korea, Seongnam, Republic of Korea) at 35 °C. Standard solutions of soyasaponins Aa, Ab, Ba, and Bb were prepared by combining 1 mg of each with 1 mL of 80% MeOH (1000 ppm). To produce four peaks in one chromatogram, identical amounts of the four standard solutions were mixed to produce a single 250 ppm solution. Each SPK extract was dissolved in 80% MeOH and sonicated for 20 min to produce a 10,000 ppm (10 mg/mL) solution. All of the solutions used in the soyasaponin tests were injected at 20 µL at 35 °C. Mobile phases A and B consisted of water containing 0.1% phosphoric acid and ACN, respectively, with a flow rate of 1.0 mL/min. The A/B ratio was set at 90:10 for 10 min, 70:30 for 20 min, 60:40 for 15 min, and finally 45:55 for 5 min. For washing and stabilization, mobile phases A and B were used at 0:100 for 8 min and 90:10 for 15 min.

### 2.12. Calibration Curves

To determine the quantity of *trans*-resveratrol in peanuts and sprouts, a calibration curve for *trans*-resveratrol was produced using seven standard solutions with different concentrations (0.98, 1.95, 3.91, 7.81, 15.62, 31.25, and 62.5 ppm). The linear equation for the calibration curve was y = 36262x − 6215.9, with strong linearity observed (*r*^2^ > 0.9999). To produce the calibration curve for soyasaponin Bb, six solutions with concentrations of 3.91, 7.81, 15.62, 31.25, 62.5, and 125 ppm were used. The linear equation was y = 2618.3x + 11129, with a linear regression coefficient (*r*^2^) of 0.9978.

### 2.13. Statistical Analysis

All experiments were run three times using the same method to ensure the reproducibility and accuracy of the results. The experimental results were expressed as the sum and difference of the mean and standard deviation. Statistical analysis was conducted using Minitab (Version 16, Minitab, Inc., State College, PA, USA). Significant differences between samples were determined using ANOVA and Tukey’s tests, with a significance level of *p* < 0.05.

## 3. Results and Discussion

### 3.1. SPK Extracts

The dry weight, extract weight, and yield of the SPK samples are summarized in Table 1. The highest yield was achieved for SPK 3 (55.0%), which produced an extract with a mixture of solid and oil. The yield of peanut extracts can depend on the solvent used for extraction (e.g., MeOH, EtOH, and water). Yu et al. reported that roasted peanut peels had the highest phenol content when extracted with 80% EtOH [28]. Extraction using EtOH has a high extraction yield, particularly when extracting bioactive compounds [29]. Therefore, in this study, high-purity 95% EtOH was used. Given that the lowest yield was 50.0% for SPK 1, it was shown that an appropriate solvent was used.

### 3.2. ABTS^+^ and DPPH Radical Scavenging Activity of SPK

Spectrophotometric ABTS^+^ and DPPH assays are widely used to evaluate redox activity based on the scavenging of free radicals and are useful indicators of antioxidant ability [30]. The ABTS^+^ and DPPH radical scavenging activity of SPK is presented in Table 2. In ABTS^+^ assays, ABTS radicals gain electrons when reduced by antioxidants, causing the blue or green ABTS^+^ to become transparent [30]. SPK 2 (6.20 mg/mL) had the highest antioxidant effect in the ABTS^+^ assays, while SPK 1 (23.32 mg/mL) exhibited the lowest antioxidant activity. When radical scavenging activity was evaluated using DPPH, SPK 3 (14.48 mg/mL) was found to have the strongest effect, while the highest IC_50_ was produced by SPK 1 (44.24 mg/mL). Thus, the antioxidant effect was strongest when the sprouts first emerged from the cotyledons, but it was almost nonexistent in the ungerminated seeds.

When comparing the results of the ABTS^+^ and DPPH assays, the ABTS^+^ assays demonstrated a higher antioxidant effect for the same samples. Floegel et al. reported that, when comparing the oxygen radical absorbance of phenolic and flavonoid compounds from 50 fruits and vegetables, the results of the ABTS+ assay showed a higher correlation with the antioxidant effect than the results of the DPPH assay [30]. Zhou et al. also reported that, when comparing the antioxidant capacity of peanut sprouts grown in different regions, ABTS^+^ assays returned higher values than DPPH assays, likely due to the relatively low reactivity of DPPH [31].

### 3.3. TPC and TFC of SPK

The TPC and TFC of the SPK extracts are presented in Table 3. Thaipong et al. reported that the three groups (e.g., vitamins, phenolics, and carotenoids) influenced the effectiveness of antioxidants [32].

The sample with the highest polyphenol content was SPK 4 (29.38 mg TAE/g ext.). In contrast, SPK 1 (16.92 mg TAE/g ext.) had the lowest polyphenol content. Considering the statistical differences, it reaffirms that the samples with good antioxidant effects had high TPC, and samples with poor antioxidant effects had low TPC. [33]. The sample with the highest flavonoid content was SPK 2 (1.61 mg QE/g ext.), which was derived from peanuts with freshly sprouted cotyledons. The sample with the lowest flavonoid content was SPK 3 (0.84 mg QE/g ext.). Overall, however, the TFC levels of the four samples were not statistically different. Peanuts contain various flavonoids, such as anthocyanins and catechins, that are powerful antioxidants.

However, because antioxidant effects are influenced by both flavonoids and non-flavonoids, TFC alone cannot accurately reflect antioxidant activity. In peanuts, in the early stages of germination, the synthesis of new flavonoids is triggered, increasing TFC. However, as germination continues, water is retained, leading to the loss of water-soluble flavonoids such as anthocyanins and catechins [31]. When sprouts reach about 1 cm in length, the TFC content decreases considerably. In addition, SPK 3 and SPK 4 are exposed to more light and oxygen as the shoot emerges from the cotyledon, and flavonoids are broken down by light and oxidized when exposed to oxygen [31]. In addition, Tran et al. reported that the content of flavonoids can vary depending on how a sample is extracted [34]. In particular, they found that EtOH was more efficient than MeOH in measuring the TFC of seeds and sprouts. However, even when EtOH was used as the extraction solvent, they reported that the TFC results still varied depending on the extraction method. In particular, the amount of TFC extracted using a reflux condenser was significantly lower than that using ultrasonic extraction. This is because reflux extraction requires heat energy to vaporize the solvent, and flavonoids can be degraded by heat. The TFC content can also vary depending on the extraction time, with longer exposure to heat leading to more flavonoids being broken down [32]. It is thus possible that, in the present study, the TFC content was reduced due to the continuous heat applied during extraction and the action of light and oxidation.

### 3.4. Content of Trans-Resveratrol

**Figure 4:**
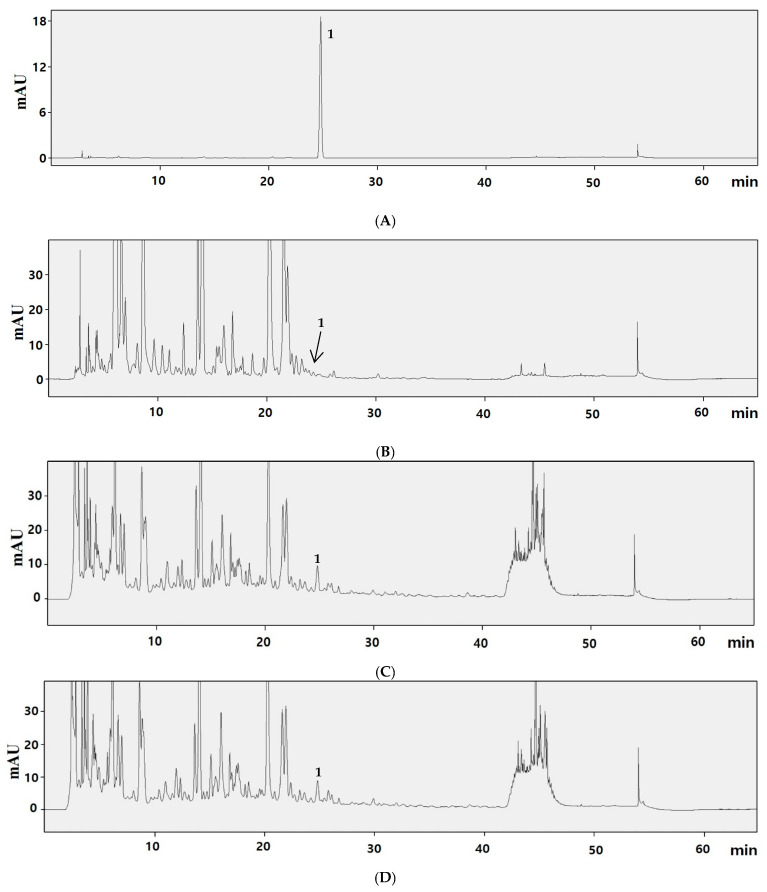

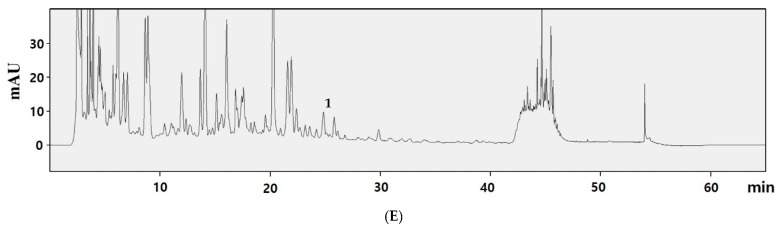
HPLC chromatograms of (**A**) *trans*-resveratrol (**1**), (**B**) SPK 1, (**C**) SPK 2, (**D**) SPK 3, and (**E**) SPK 4.

**Figure 5:**
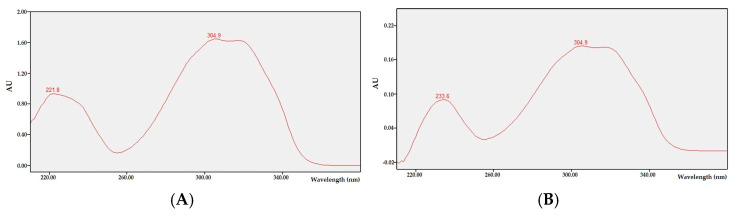
UV spectrums of (**A**) *trans*-resveratrol and (**B**) SPK 2.

MS and NMR data were not available, as *trans*-resveratrol was not isolated from the sample. Instead, the presence of *trans*-resveratrol in peanuts is supported by the previously published literature [2,10,11,18]. Within the UV spectrum (210–400 nm), *trans*-resveratrol has a high absorbance at 310 nm. Based on this, HPLC was performed at a wavelength of 310 nm to determine the absorbance of a 50,000 ppm *trans*-resveratrol standard. A shoulder-less and symmetrical peak was subsequently found at 24.80 min. When screening the chromatograms of the SPK samples, a single peak at 24.80 min was observed (Figure 4). Based on the area of the peak, the sample with the highest *trans*-resveratrol content was SPK 2 (0.054 mg/g ext.). In contrast, only trace amounts of *trans*-resveratrol were detected in SPK 1 (Table 4). To obtain additional data supporting the presence of *trans*-resveratrol, UV spectrums of the standard and samples were compared, and it was confirmed that the UV absorption wavelengths of *trans*-resveratrol and the samples of SPK were comparable (Figure 5).

In another study, the *trans*-resveratrol content of raw seeds of Palkwang, one of the parent varieties of SPK, was found to be 0.14 µg [35]. Although the exact values cannot be compared in this study, the *trans*-resveratrol content in SPK after sprouting increased more than 100-fold compared to that in raw seeds of Palkwang. Pae et al. found that the average *trans*-resveratrol content after germination of 37 peanut cultivars in Korea was 43.9 µg, and the *trans*-resveratrol content of Palkwang after germination was 45.8 µg. [36]. The content of *trans*-resveratrol in SPK after germination was also detected similarly.

We hypothesized that, as the shoots grow, more *trans*-resveratrol is generated. There are two possibilities as to why the content of *trans*-resveratrol decreases as peanut sprouts grow: either it is decomposed, or it is used. The structure of resveratrol changes when it reacts with light, and this is more apparent under more intense UV light [37]. Specifically, when *trans*-resveratrol is exposed to UV-B radiation, most of it transforms into other forms within 4 h. However, under laboratory conditions, which primarily involve visible light, *trans*-resveratrol remains stable [38]. Since the seeds were germinated in darkness, and the experiments were conducted indoors, light exposure likely had a minimal effect on *trans*-resveratrol content. Additionally, one study showed that *trans*-resveratrol influences the growth of lettuce aerial parts [39], suggesting that *trans*-resveratrol may have been utilized during the growth of peanut sprouts.

### 3.5. Content of Soyasponins Aa, Ab, Ba, and Bb

**Figure 6:**
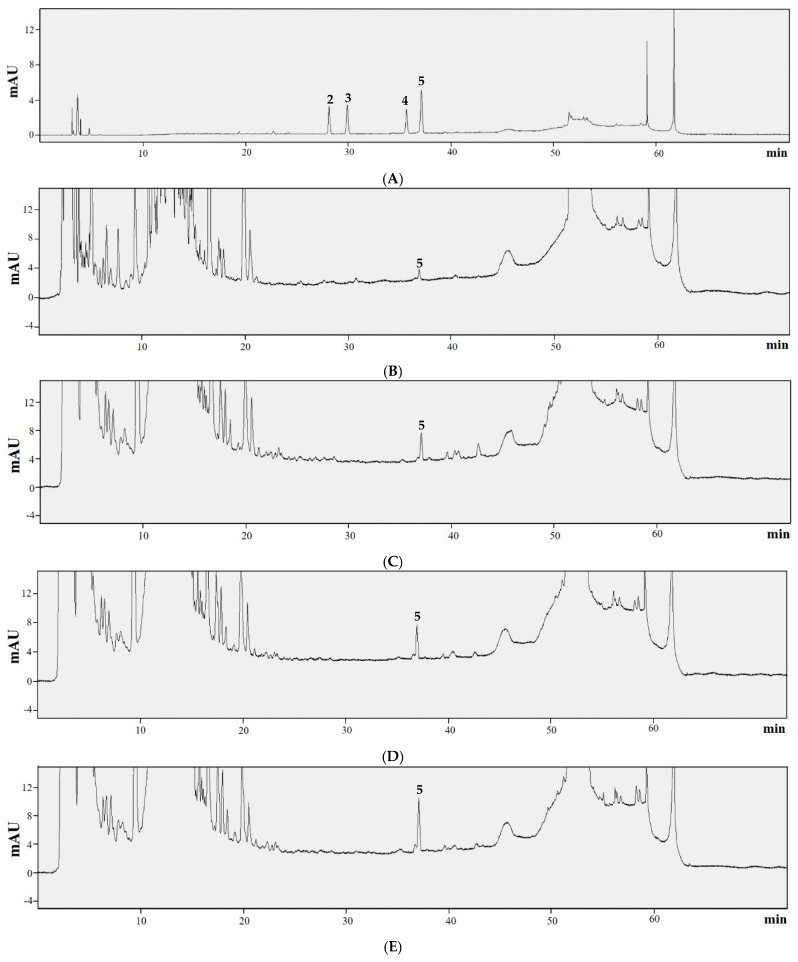
HPLC chromatograms for (**A**) soyasaponins Aa (**2**), Ab (**3**), Ba (**4**), Bb (**5**), (**B**) SPK 1, (**C**) SPK 2, (**D**) SPK 3, and (**E**) SPK 4.

MS and NMR data were not available, as soyasaponin Bb was not isolated from the sample. Instead, the presence of soyasaponin Bb in peanuts is supported by the previously published literature [40,41]. Within the UV spectrum (190–400 nm), soyasaponin group A and B show high absorbance near 210 nm. Because of the low absorption wavelength below 210 nm, UV spectral data were not sufficient to confirm the presence of soyasaponin Bb. Therefore, a spike test was conducted to observe the change in the HPLC chromatogram by adding soyasaponin Bb standard to the SPK sample. When the SPK samples and soyasaponin Bb standard were mixed, the peak area increased, and no new peaks were discovered. Additionally, to confirm the presence of other types of soyasaponin, four standards of soyasaponin, Aa, Ab, Ba, and Bb, were mixed and analyzed by HPLC. The peak of soyasaponin Bb with a retention time of 37.9 min was observed in all SPK samples. However, the peaks for soyasaponin Aa, Ab, and Ba with retention times of 29.5, 31.3, and 36.5 min were not detected in all SPK samples (Figure 6).

The content of soyasaponin Bb was found to be highest in the SPK 4 extract, which was taken from the longest shoots, but was the lowest in the SPK 1 extract, with a 6.5-fold difference between the two. Similarly, in a study analyzing germinated black beans, the content of soyasaponin Bb was found to increase by approximately 40 mg/100 g after germination [42]. In another study, the content of soyasaponin Bb in soybeans before and after germination was compared, with a 15-fold difference observed between the two [43]. These studies and the results of the present study suggest that soyasaponin Bb is continuously synthesized and accumulates during the germination process [43].

**Figure 7:**
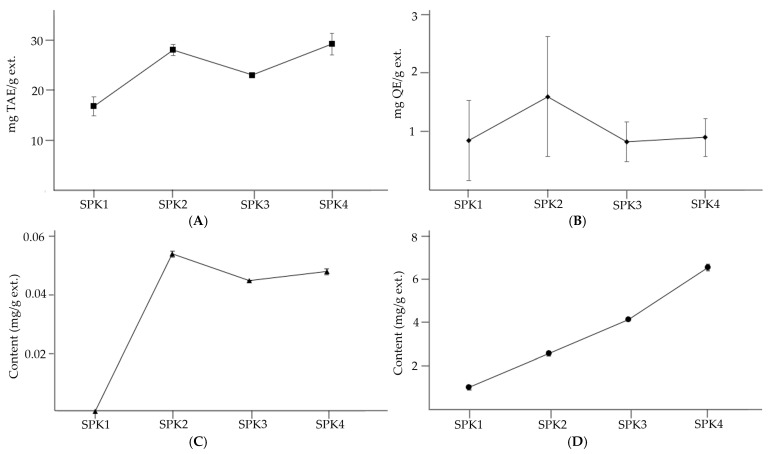
Tendency of SPK for (**A**) TPC, (**B**) TFC, (**C**) content of *trans*-resveratrol, and (**D**) soyasaponin Bb.

Soyasaponin Bb is a photo-response compound whose content in soybeans changes significantly depending on the presence of light [44]. For example, the soyasaponin Bb content is higher when soybean seeds germinate under light than in the dark [44]. The findings from the experiments conducted in this study are summarized in the following graph (Figure 7).

## 4. Conclusions

This study showed that germination had a positive effect on increasing the nutritional value of seeds. The germinated seeds showed high antioxidant effects and increased phenolic compounds and phytochemical nutrients. Our study confirmed that germination, a low-cost and practical method, is very effective in improving nutritional value. SPK2 exhibited the highest TFC (1.61 mg QE/g ext.) and *trans*-resveratrol content (0.054 mg/g ext.), while SPK4 showed the highest TPC (29.38 mg TAE/g ext.) and soyasaponin Bb content (6.543 mg/g ext.). In terms of radical scavenging activities, SPK2 and SPK3 performed best in the ABTS^+^ and DPPH assays, respectively. Germinated samples demonstrated strong results across all analyses, highlighting the benefits of germination in enhancing phytochemical properties. The increased bioactive compounds through germination and the high production of newly bred SPK have the potential for wide application in nutritional and therapeutic aspects. Further research can be conducted to determine appropriate germination conditions that induce the desired nutritional compositions.

## Figures and Tables

**Table 1:** Extract yield of SPK samples.

Sample	DW (g)	Ext. Weight (g)	Yield (%)
SPK 1	6	3.0	50.0
SPK 2	6	3.1	51.7
SPK 3	6	3.3	55.0
SPK 4	6	3.2	53.3

Note: DW, dry weight; Ext., extract.

**Table 2:** ABTS^+^-DPPH radical scavenging activity of SPK extracts.

Sample	IC_50_ (mg/mL)	
	ABTS^+^	DPPH
SPK 1	23.32 ± 0.30 ^a^	44.24 ± 0.66 ^a^
SPK 2	6.20 ± 0.08 ^d^	17.61 ± 0.80 ^b^
SPK 3	11.75 ± 0.02 ^b^	14.48 ± 0.87 ^c^
SPK 4	7.63 ± 0.08 ^c^	15.05 ± 0.71 ^c^
Ascorbic acid	0.11 ± 0.00	0.13 ± 0.00

Note: ^a–d^ different letters in the same column indicate significant statistical differences (*p* < 0.05).

**Table 3:** TPC and TFC of SPK extracts.

Sample	TPC (mg TAE/g ext.)	TFC (mg QE/g ext.)
SPK 1	16.92 ± 1.93 ^c^	0.86 ± 0.69 ^a^
SPK 2	28.22 ± 1.11 ^a^	1.61 ± 1.03 ^a^
SPK 3	23.16 ± 0.04 ^b^	0.84 ± 0.34 ^a^
SPK 4	29.38 ± 2.18 ^a^	0.91 ± 0.32 ^a^

Note: ^a–c^ different letters in the same column indicate significant statistical differences (*p* < 0.05). TAE, tannic acid equivalent; QE, quercetin equivalent.

**Table 4:** Content of *trans*-resveratrol and soyasaponins of SPK extracts.

Sample	Content (mg/g ext.)					
	1	2	3	4	5	Total
SPK 1	Trace	ND	ND	ND	1.025 ± 0.102 ^d^	1.025
SPK 2	0.054 ± 0.001 ^a^	ND	ND	ND	2.587 ± 0.102 ^c^	2.641
SPK 3	0.045 ± 0.000 ^c^	ND	ND	ND	4.149 ± 0.045 ^b^	4.194
SPK 4	0.048 ± 0.001 ^b^	ND	ND	ND	6.543 ± 0.146 ^a^	6.591

Note: ^a–d^ different letters in the same column indicate significant statistical differences (*p* < 0.05). ext., extract. **1**: *trans*-resveratrol, **2**: soyasaponin Aa, **3**: soyasaponin Ab, **4**: soyasaponin Ba, and **5**: soyasaponin Bb.

## Data Availability

The data presented in this study are available on request from the corresponding author.
